# Resveratrol Confers Protection against Rotenone-Induced Neurotoxicity by Modulating Myeloperoxidase Levels in Glial Cells

**DOI:** 10.1371/journal.pone.0060654

**Published:** 2013-04-08

**Authors:** Chi Young Chang, Dong-Kug Choi, Dae Kee Lee, Young Jun Hong, Eun Jung Park

**Affiliations:** 1 Cancer Immunology Branch, National Cancer Center, Goyang, Korea; 2 Division of Life and Pharmaceutical Sciences, The Graduate School of Ewha Woman’s University, Seoul, Korea; 3 Department of Biotechnology, College of Biomedical & Health Science, Konkuk University, Chungju, Korea; 4 Department of Laboratory Medicine, Korea Cancer Center Hospital, Seoul, Korea; Virginia Commonwealth University, United States of America

## Abstract

Myeloperoxidase (MPO) functions as a key molecular component of the host defense system against diverse pathogens. We have previously reported that increased MPO levels and activity is a distinguishing feature of rotenone-exposed glial cells, and that either overactivation or deficiency of MPO leads to pathological conditions in the brain. Here, we provide that modulation of MPO levels in glia by resveratrol confers protective effects on rotenone-induced neurotoxicity. We show that resveratrol significantly reduced MPO levels but did not trigger abnormal nitric oxide (NO) production in microglia and astrocytes. Resveratrol-induced down-regulation of MPO, in the absence of an associated overproduction of NO, markedly attenuated rotenone-triggered inflammatory responses including phagocytic activity and reactive oxygen species production in primary microglia and astrocytes. In addition, impaired responses of primary mixed glia from *Mpo* ^−/−^ mice to rotenone were relieved by treatment with resveratrol. We further show that rotenone-induced neuronal injury, particularly dopaminergic cell death, was attenuated by resveratrol in neuron-glia co-cultures, but not in neurons cultured alone. Similar regulatory effects of resveratrol on MPO levels were observed in microglia treated with MPP^+^, another Parkinson’s disease-linked neurotoxin, supporting the beneficial effects of resveratrol on the brain. Collectively, our findings provide that resveratrol influences glial responses to rotenone by regulating both MPO and NO, and thus protects against rotenone-induced neuronal injury.

## Introduction

Myeloperoxidase (MPO) is an oxidant-generating enzyme that catalyzes the formation of the potent oxidant hypochlorous acid (HOCl) and other chlorinating species derived from hydrogen peroxide (H_2_O_2_) [1], [2]. MPO and MPO-derived oxidants could mediate inflammatory responses at sites of inflammation, thereby contributing to the defense system against pathogens [3]. Increasing evidence has suggested potential links between MPO and the development of disease such as ischemia, atherosclerosis, and acute myeloid leukemia [4]–[6]. Studies have shown that blockade of MPO activity in mouse models and in humans can reduce pathological responses in diseases such as renal ischemia [4], [7]. By contrast, it has been reported that MPO deficiency can also lead to pathological conditions in animal models and patients. There are reports that MPO-deficient mice have a significantly increased incidence of inflammation-associated diseases including experimental autoimmune encephalomyelitis, atherosclerosis, and lung inflammation [8], [9]. Similarly, MPO-deficient patients have been shown to have a greater occurrence of severe infections and chronic inflammatory processes [10]. Recently, we also found that glial cells from MPO-knockout (*Mpo^−/−^*) mice exhibit marked pathological properties under rotenone-exposed conditions [11]. Collectively, these findings strongly suggest that an appropriate level of MPO may be important for preventing the development of MPO-associated inflammatory diseases. However, the precise roles of MPO in this context and the underlying mechanisms responsible for its action have not been fully determined.

Resveratrol (*trans*-3, 4′,-5-trihydroxystilebene), a natural phytoalexin compound found in various plants such as grapes and berries, is known to have potent anti-oxidant and anti-tumorigenic activities [12]–[15]. The beneficial effects of resveratrol are reported to be closely associated with its anti-inflammatory properties, including modification of eicosanoids synthesis [13], [15], [16]. In addition, there are reports that resveratrol increases the activity of manganese superoxide dismutase (Mn-SOD), thereby conferring resistance to mitochondrial dysfunction, permeability transition, and apoptotic death, in various disease states [17], [18]. Moreover, studies have shown that resveratrol protects against neuronal cell dysfunction and death, and may be of assistance in the treatment of neurodegenerative diseases such as Huntington’s, Alzheimer’s, and Parkinson’s disease (PD) [19]–[21]. Evidence accumulated to date suggests the clinical potential of resveratrol, but the precise molecular mechanism for its biological activities in the brain remains poorly understood.

Rotenone, one of the most widely used pesticides, has been suggested to be the primary environmental risk factor of chronic inflammatory diseases like PD [22]–[24]. Chronic rotenone exposure causes a systemic defect in mitochondrial complex I and oxidative stress, contributing to the pathogenesis of PD [25]. We previously reported the novel finding that MPO may be associated not only with pathological outcomes, but also with protective events in brain-resident immune cells under rotenone-exposed conditions [11]. Our studies with MPO inhibitors and *Mpo^−/−^* mice revealed that MPO deficiency potentiates, rather than inhibits, the rotenone-induced activated state of glia, thereby leading to abnormal outcomes in glia and neurons. MPO deficiency leads to an augmentation in rotenone-induced ROS generation in microglia, and impairs the regulatory system of glial cells against rotenone exposure in the brain. MPO has been reported to act as a direct and significant mediator of decreased NO-bioavailability [2]. MPO can oxidize NO, thereby inhibiting NO-dependent signaling and modulating redox-sensitive signaling cascades during inflammation [26], [27]. In this regard, we hypothesized that aberrant generation of ROS in MPO-deficient glia could be due, at least in part, to increased NO bioavailability, and asked whether a decrease in MPO that was not accompanied by excessive ROS generation would be helpful in protecting against pathological consequences of rotenone exposure in the brain. In the present study, we demonstrate that down-regulation of MPO in microglia and astrocytes without leading to overproduction of nitric oxide (NO) effectively protects rotenone-exposed neurons, and as such could be a promising therapeutic strategy for ameliorating rotenone-triggered pathological events in the brain.

## Materials and Methods

### Reagents and Antibodies

Rotenone and human MPO were obtained from Calbiochem (La Jolla, CA). The antibodies used in this study included mouse anti-α-tubulin (Sigma-Aldrich, St. Louis, MO), anti-MPO (Dako, Denmark), anti-gp91 phox (Abcam, Cambridge, MA), and anti-tyrosine hydroxylase (anti-TH; Abcam, Cambridge, MA). Fluorophore-conjugated secondary antibodies (Alexa Fluor 488) were purchased from Molecular Probe (Eugene, OR).

### Animals

Sprague-Dawley (SD) rats and adult timed-pregnant SD rats were purchased from ORIENT BIO (Sungnam, Korea). C57BL/6J and B6.129X1-MPO*^tm1Lus^*/J mice were purchased from The Jackson Laboratory. All animal procedures were performed according to the National Cancer Center guidelines for the care and use of laboratory animals. The protocol was approved by the Committee on the Ethics of Animal Experiments of the National Cancer Center (Permit Number: NCC-08-031). Tissues isolated from the organs after the anesthesia with Zoletil/Rompun mixture (ratio, 4∶1) or euthanasia were prepared and analyzed in an optical microscope.

### Primary Microglia, Astrocytes, and Mixed Glial Cell Cultures

Glial cells isolated from the cerebral cortex of 1- to 3-day-old SD rats or B6.129X1-MPO*^tm1Lus^*/J mice were triturated into single-cell suspensions, plated in 75-cm^2^ T-flasks and cultured in MEM containing 10% FBS for 2 weeks. The microglia and astrocytes were prepared as described in our previous studies [11], [28]. The proportion of microglia in murine mixed glial cultures was demonstrated to be 30 to 50 percent by FACS analyses using anti-CD11b antibody. Murine BV2 microglial cells were maintained in DMEM supplemented with 5% FBS, 100 U/ml penicillin and 100 µg/ml streptomycin at 37°C in a humidified incubator under 5% CO_2_.

### Neuron-enriched Mesencephalic Cultures

Ventral mesencephalic tissues were dissected from embryonic day 14 or 15 SD rats and dissociated enzymatically (0.1% trypsin) and mechanically [29]. Rat neurons were cultured as described in our previous study [11]. Cells were seeded onto 6 well plates (2×10^6^ cells/well) or 24 well plates (5×10^5^ cells/well) pre-coated with poly-D-lysine (5 mg/ml) and laminin (0.2 mg/ml). Of the mesencephalic cultures, approximately 3% were TH-positive neurons by immunocytochemistry using anti-TH antibody (Calbiochem), as reported by Gao *et al.* [30]. For neuron-microglia co-culture, rat primary mesencephalic neuron-enriched cultures were seeded in 24 well plates, and incubated with rat primary microglia using transwell chambers (BD Falcon, 0.4 µm pore size). The co-cultures were established by adding 10% (5×10^4^/transwell insert) of microglia to neuron-enriched cultures in the chemically defined serum-free medium.

### Phagocytosis Assay

Microglia were plated in 60-mm^2^ dishes (2.5×10^4^ cells/dish) and then treated or left untreated with rotenone in the presence or absence of 5% FBS. Phagocytic capacity was measured as described in our previous study [11], [28].

### MPO Activity Assay

MPO activity was measured using an EnzChek® Myeloperoxidase Activity Assay Kit as described by the manufacturer (Molecular Probes, Invitrogen, CA, USA). Fluorescence was measured at 590 nm following excitation at 530 nm using a Spectra-Max Gemini fluorometer (Molecular Devices, CA, USA) at room temperature.

### Measurements of NO Release

The media nitrite concentration was measured as an indication of NO release. Following the indicated cell incubations, 50 µl of culture medium was removed and mixed with an equal volume of Griess reagent (0.1% naphthylethylene diamine, 1% sulfanilamide, 2.5% H_3_PO_4_), and the absorbance of the mixture was measured at 540 nm.

### Confocal Microscopy

Cells grown on cover slips were fixed in ice-cold methanol and incubated with the primary antibodies (1∶300 dilution for anti-MPO and anti-TH) for overnight at 4°C. Fluorescent images were acquired with a confocal laser scanning microscopy system (CLSM; model LSM510 meta; Carl zeiss, Jena, Germany). The confocal system software was used to capture and store the images.

#### Reverse Transcriptase-Polymerase Chain Reaction (RT-PCR) and quantitative real-time PCR analyses

Total RNA was isolated using Easy-Blue (iNtRON, Korea), and cDNA was synthesized using avian myeloblastosis virus reverse transcriptase (TaKaRa, Japan) according to the manufacturer’s instructions. The sequences of PCR primers were previously reported [11]. The QuantiFast SYBR Green PCR kit (Qiagen, German) was used for real-time PCR. Roche LightCycler 480 Real-Time PCR System (Roche applied science, UK) and LigthCycler 480 Quantification Software Version 1.5 were used for real time PCR performance and analysis. The sequences for real time PCR are as follows; rat COX-2 forward primer 5′- TGTATGCTACCATCTGGCTTCGG, reverse primer 5′-GTTTG GAACAGTCGCTCGTCATC; rat GAPDH forward primer 5′-TGGAGAAACCTGCCAAG TATGA, reverse primer 5′- TGGAAGAATGGGAGTTGCTGT; rat iNOS forward primer 5′- CTTGGAAGAGGAACAACTACTGCT, reverse primer 5′- GCCAAATACCGCATACCTGA A; rat TNF-α forward primer 5′- TGTCTGTGCCTCAGCC TCTTC, reverse primer 5′- TTTGGGAACTTCTCCTCCTTGT.

### Western Blot Analysis

Cells were lysed in ice-cold modified RIPA buffer [11], and the lysate was separated by sodium dodecyl sulfate-polyacrylamide gel electrophoresis (SDS-PAGE) on 10% gels and transferred to nitrocellulose membranes. The membranes were incubated with primary antibodies (anti-MPO, 1∶3000 dilution; anti-gp91, 1∶1000 dilution; anti-tubulin, 1∶3000), and then visualized using an enhanced chemiluminescence (ECL) system. Quantification of western blot results using the band densitometry analysis performed with Image J software.

### Flow Cytometric Analysis

For CD16/32 surface stain, cells were incubated anti-CD16/32 antibody (BD, 1∶300 dilution) and goat anti-rat Alexa488 secondary antibodies (Molecular Probe, 1∶1000 dilution) for 20 min at 4°C. For intracellular MPO level, cells were fixed in 4% paraformaldehyde in PBS for 15 min and incubated with the permeable buffer (eBioscience). Primary antibody incubations (1∶1000 dilution for anti-MPO) and goat anti-rabbit Alexa488 secondary antibody incubations (1∶1000 dilution) were performed for 20 min at 4°C. Flow cytometric measurements were performed using a Becton Dickinson FACSCalibur system and data were analyzed using FlowJo software (Treestar, Inc., San Carlos, CA).

### Measurement of Reactive Oxygen Species

Cells were suspended in 5 µM CM-H_2_DCFDA (DCF; Molecular Probes, Eugene, OR) for 30 min at 37°C in dark and suspended in PBS. The green emission of DCF was measured using a FACSCalibur (BD). DCF detects H_2_O_2_, hydroxyl radical, peroxyl radical, and peroxynitrite anion.

### Lactate Dehydrogenase Assay

Lactate dehydrogenase (LDH) released into the supernatant by damaged cells was measured according to previous report [11] and determined by measuring absorbance at a wavelength of 340 nm in kinetics mode for 5 min on a microplate reader (Molecular Devices). Percent cytotoxicity was calculated as follows using total cellular LDH as a low control: Cytotoxicity (%) = [(experimental value – low control)/(high control – low control)]×100%.

### Cell Viability Assay

Cell viability was determined using the Live/Dead Viability Cytotoxicity Kit (Molecular Probes, Eugene, OR) or Cell Counting Kit-8 (CCK-8, Dojindo Laboratories, Japan) according to the manufacturer’s instruction and our previous reports [11], [31].

### Tyrosine Hydroxylase Labeling and Morphological Analysis

Mesencephalic cultures were plated on to coverslip pre-coated with poly-D-lysine and laminin. For morphological analysis, mesencephalic cultures were very carefully fixed with 4% paraformaldehyde, and then incubated at 4°C in PBS containing a 1∶300 dilution of the anti-TH antibody (Abcam, Cambridge, MA). Digital images were acquired with using Axio Observer Z1 (Carl Zeiss) and morphological measurements were performed using AxioVision software (Carl Zeiss). For morphological analysis, the number of primary dendrites and average maximal length of the dendrites were calculated from morphological structures outlined during the analysis. Maximal dendrite length was defined as the distance from the soma to the tip of the longest dendrite for each neuron. The number of primary dendrites was determined as those dendrites directly stemming from the soma. The number of TH-positive neurons per coverslide was counted using a 20× objective and more than 20 frames for each coverslide were examined [32].

### Data Analysis

All data were expressed as the mean ± SD and all statistical analyses were performed using the Statistical Analysis System (SAS) software version 9.1.3 (SAS Institute, Cary, NC). The statistical significance was calculated using one-way ANOVA and the results were presented in the Figure legends and (Table S1). Results were considered statistically significant when *P<0.05*.

## Results

### Resveratrol Reduces both MPO Levels and NO Production in Rotenone-exposed Microglia

**Figure 1:**
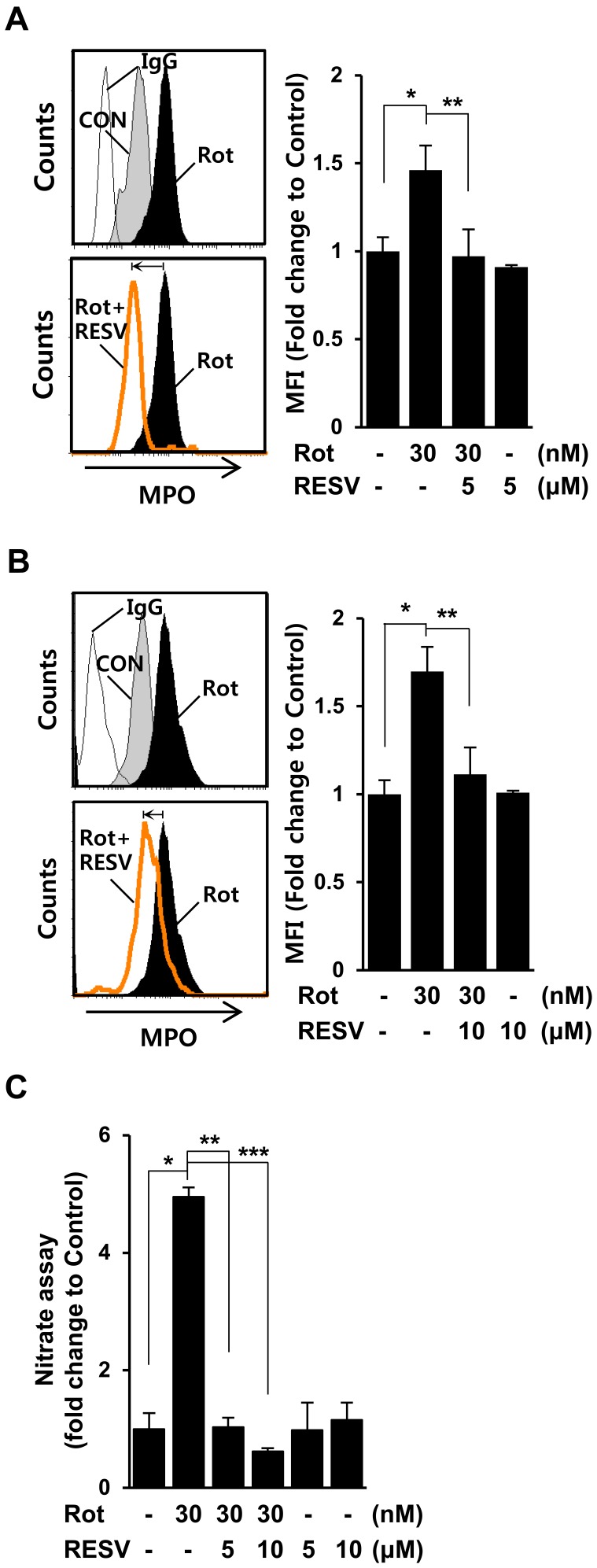
Resveratrol suppresses both MPO and NO production in rotenone-exposed microglia. **A–B.** Mouse BV2 microglial cells (**A**) and rat primary microglia (**B**) were pre-treated with or without the indicated concentrations of resveratrol (RESV) for 1 h, and then incubated with 30 nM rotenone (Rot) for 24 h. The levels of MPO were analyzed by FACS using an anti-MPO antibody. The graph represents the fold changes in MFI (mean fluorescence intensity) ± SD from more than three independent experiments. The graph represents the fold changes in Mean ± SD of three independent experiments. *P<0.05, **P<0.01 compared with rotenone-treated cells. **C.** Rat primary microglia were mock-treated or treated with 30 nM rotenone in the presence or absence of the indicated concentrations of resveratrol for 24 h, and supernatants were assayed for nitrate concentration. *P = 0.01; **P = 0.001; ***P = 0.001.

Our recent report suggested that MPO triggers pro-inflammatory responses in glia under rotenone-exposed conditions, but showed that an MPO deficiency further exacerbates rotenone-induced generation of reactive oxygen species (ROS) such as NO in microglia and astrocytes, thereby increasing neuronal cell death [11]. Therefore, we searched for candidate agents that not only reduced abnormal MPO activity but also decreased excessive NO production in microglia under rotenone-exposed conditions. In a screening of anti-inflammatory agents, we found that resveratrol significantly reduced both MPO levels and NO release in rotenone-exposed microglia. Mouse BV2 microglial cells were pre-treated with 5 µM resveratrol for 1 h, and then mock-treated or treated with 30 nM rotenone, after which intracellular MPO levels were determined by FACS analyses using an anti-MPO antibody. As shown in Fig. 1A, rotenone-induced MPO levels were significantly suppressed by pretreatment with resveratrol. Similar results were observed in rat primary microglia (Fig. 1B). In addition, the rotenone-triggered release of NO was markedly attenuated by pretreatment of rat primary microglia with the indicated concentrations of resveratrol (Fig. 1C). These findings indicate that resveratrol may be capable of reducing not only MPO levels but also NO release in microglia under rotenone-exposed conditions.

### Resveratrol Directly Suppresses MPO Levels and Activity in both Rat Primary Microglia and Astrocytes

**Figure 2:**
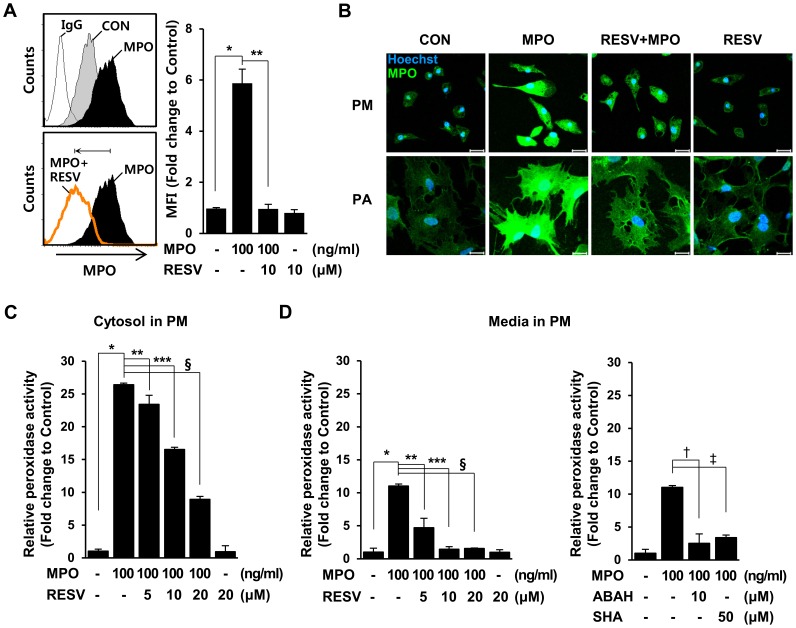
Resveratrol reduces MPO levels and its activity in MPO-treated rat primary microglia and astrocytes. **A.** Rat primary microglia were pre-treated with or without 10 µM resveratrol for 1 h, and then treated with 100 ng/ml MPO for 24 h, after which MPO levels were determined by FACS analyses. The graph represents the fold changes in MFI ± SD from three independent experiments. *P = 0.027; **P = 0.049. **B.** Rat primary microglia (PM, upper) and astrocytes (PA, lower) were pretreated with 10 µM (PM) or 20 µM (PA) of resveratrol for 1 h. The cells were incubated with 100 ng/ml MPO for 24 h and MPO levels were observed by confocal microscopy using an anti-MPO antibody. MPO, green; DAPI, blue; scale bar = 20 µm. The data shown are representative of at least three independent experiments. **C–D.** Primary microglia were mock-treated or treated with 100 ng/ml MPO in the presence of the indicated concentrations of resveratrol, 4-aminobenzoylhydrazide (ABAH), or salichylhydroxamic acid (SHA). Total cell lysates (**C**) and media (**D**) were collected, and then MPO activity was measured fluorometrically (SpectraMax Gemini EM spectrofluorometer, Molecular Devices) using an EnzChek® Myeloperoxidase activity assay kit. The results are the fold changes in Mean SD of three experiments performed in triplicate. *P<0.05, **P<0.01, ***P<0.001 compared with MPO-treated cells.

Having shown that resveratrol significantly reduced rotenone-stimulated MPO levels and prevented the accompanying overproduction of NO in microglia, we asked whether resveratrol directly reduced MPO levels in MPO-treated rat primary microglia. Cells were left untreated or pretreated with 10 µM resveratrol for 1 h, and then mock-treated or treated with 100 ng/ml MPO for 24 h. FACS analyses showed that MPO-dependent increases of MPO levels in rat primary microglia were markedly inhibited by pretreatment with resveratrol (Fig. 2A). Immunocytochemistry also showed the inhibitory effects of resveratrol on MPO levels in rat primary microglia (Fig. 2B). Because a recent report suggested that resveratrol differentially modulates some inflammatory responses of microglia and astrocytes [33], we further examined the effect of resveratrol on MPO levels in rat primary astrocytes. Similar to the results obtained in microglia, resveratrol also markedly reduced MPO levels in rat primary astrocytes (Fig. 2B and data not shown).

MPO is an enzyme that catalyzes the formation of the potent oxidant HOCl and other chlorinating species derived from H_2_O_2_ [1], [2]. Thus, we further examined whether resveratrol could influence MPO activity in glial cells using a peroxidase activity-based colorimetric MPO activity assay kit. Rat primary microglia were mock-treated or treated with MPO in the presence of the indicated concentrations of resveratrol. Whole-cell lysates and media were collected, and MPO activity was measured using an EnzChek MPO activity assay kit. As shown in Fig. 2C, intracellular MPO peroxidase activity was significantly suppressed by resveratrol in a concentration-dependent manner; a similar resveratrol concentration-dependent decrease in MPO activity was also observed in media, and the extent of the inhibitory effect of resveratrol was similar to that of representative MPO inhibitors, 4-aminobenzoylhydrazide (ABAH) and salicylhydroxamic acid (SHA) (Fig. 2D). Similar results were obtained in rat primary astrocytes (data not shown). These results indicate that resveratrol directly reduces MPO levels and activity in both microglia and astrocytes.

### Both Pretreatment and Post-treatment with Resveratrol Effectively Suppress MPO Expression

**Figure 3:**
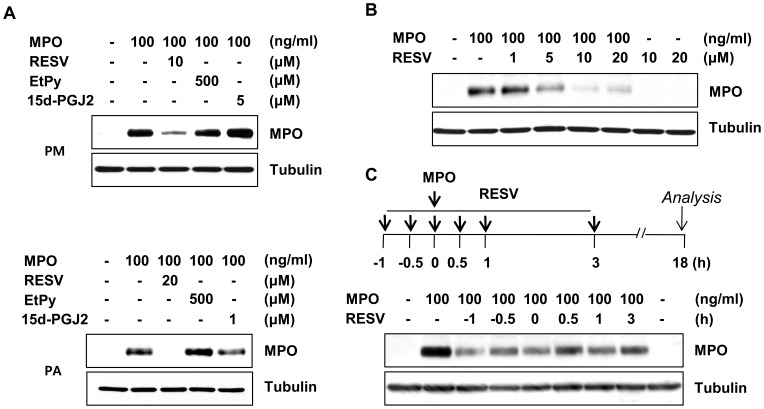
MPO-dependent increase of MPO levels are attenuated by resveratrol in rat primary microglia and astrocytes. **A.** Rat primary microglia (PM, upper) and astrocytes (PA, lower) were pretreated with resveratrol (RESV), ethyl pyruvate (EtPy), or 15d-PGJ2 for 1 h, followed by incubation with 100 ng/ml MPO for 18 h. MPO levels were determined by Western blot analyses. **B.** Concentration-dependent effects of resveratrol were observed in MPO-treated primary microglia. **C.** Rat primary microglia were treated with 20 µM resveratrol and/or 100 ng/ml MPO at various time points. The cells were further incubated for 18 h and then MPO levels were examined by western blot analyses. The data shown are representative of at least three independent experiments.

MPO is able to augment its own expression and activity in microglia and astrocytes. Accordingly, we investigated whether resveratrol could influence MPO expression in rat primary microglia and astrocytes treated with MPO. Western blot analyses showed that resveratrol specifically suppressed the MPO-induced increase in MPO expression in both primary microglia and primary astrocytes (Figs. 3A & 3B; Figure S1A & S1B). However, we did not observe any significant decrease of MPO in the presence of other representative anti-inflammatory drugs, namely ethyl pyruvate (EtPy) and 15-deoxy-delta-12,14-prostaglandin J2 (15d-PGJ2).

To further address the suppressive action of resveratrol on MPO expression, we tested the effects of resveratrol on microglia after treatment with MPO. For this, we added resveratrol into rat primary microglia at various times, from 1 h pretreatment to 3 h after treatment with MPO, and then incubated microglia for an additional 18 h. Intriguingly, the suppressive effects of resveratrol on MPO expression were observed at all treatment times tested (Figs. 3C & Figure S1C). Collectively, these results demonstrate that resveratrol can suppress MPO levels in microglia and astrocytes, even at times after treatment with MPO, suggesting the possibility that resveratrol could be an efficient agent in pathological MPO-overexpressing settings, such as the rotenone-exposed brain.

### Decreased MPO Level in the Absence of Accompanying Overproduction of NO Significantly Attenuates Rotenone-triggered Inflammatory Responses in Microglia

**Figure 4:**
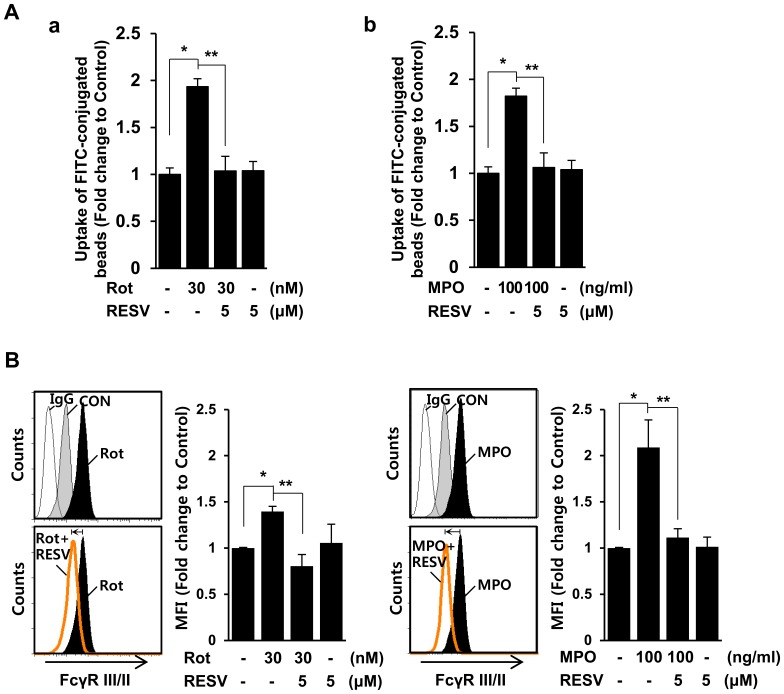
Either rotenone- or MPO-stimulated increase of phagocytic activity of microglia was markedly reduced by resveratrol. A. BV2 microglia were treated with 30 nM rotenone (Rot, *a*) or 100 ng/ml MPO (*b*) in the presence of 5 µM resveratrol (RESV) for 24 h, and the cellular uptake of FITC-conjugated fluorescent beads was determined by flow cytometric analyses. The graph represents the fold changes in MFI ± SD from three independent experiments. *P<0.05, **P<0.01, ***P<0.001 when compared with rotenone-or MPO-treated cells. B. BV2 microglia were pretreated with 5 µM resveratrol for 1 h, and followed by exposure to 30 nM rotenone (left) or 100 ng/ml MPO (right) for 24 h. The expression of Fcγ receptors was determined by FACS analyses using an anti-CD16/CD32 antibody directed against FcγIII/II receptors. The graph represents the fold changes in MFI ± SD from three independent experiments. *P<0.05, **P<0.01, ***P<0.001 when compared with rotenone-or MPO-treated cells.

Next, we asked whether resveratrol-induced down-regulation of MPO in the absence of an increase in NO could attenuate rotenone-stimulated inflammatory responses in microglia. We first examined the phagocytic activity of microglia, a representative characteristic property of activated microglia under pathological conditions. FACS analyses showed that the rotenone-stimulated increase of the cellular uptake of fluorescent beads was considerably suppressed by treatment with resveratrol (Fig. 4Aa). In addition, the MPO-induced increase in phagocytic activity was also reduced by resveratrol (Fig. 4Ab). We observed the effects of resveratrol on phagocytic activity of BV2 microglia in both the presence and absence of serum (Fig. 4A and data not shown, respectively). Next, we examined the expression level of Fcγ receptors since Fcγ receptors on BV2 microglial cells contribute to diverse cellular functions and FcγRs-mediated phagocytosis is associated with inflammatory activity of microglia [34], [35]. FACS analyses using a rat anti-mouse CD16/CD32 antibody revealed that increase in Fcγ receptors, whether induced by rotenone or MPO, were considerably suppressed by co-treatment with resveratrol (Fig. 4B). These results indicate that resveratrol attenuates the phagocytic activity of microglia under MPO- or rotenone-exposed conditions.

**Figure 5:**
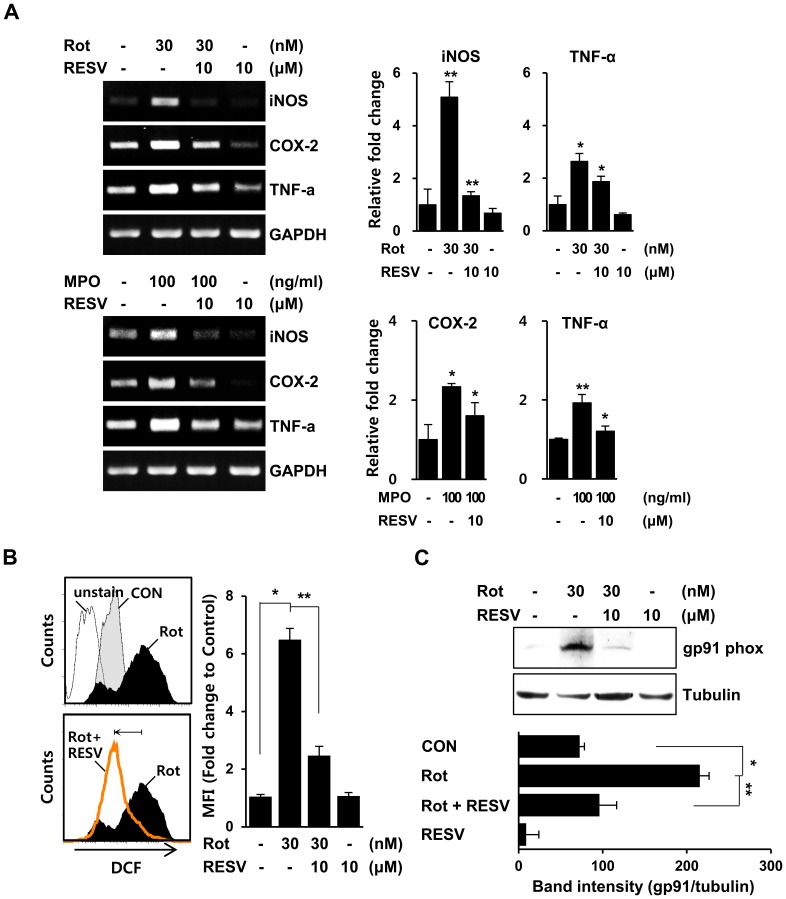
Resveratrol attenuates the expression of inflammation-associated genes and ROS under rotenone- or MPO-exposed microglia. **A.** Primary microglia were stimulated with rotenone (upper) or MPO (lower) in the presence or absence of resveratrol, after which the mRNA levels of iNOS, COX-2, and TNF-α were determined by RT-PCR (left) and quantitative real-time PCR analyses (right). The data are representative of three independent experiments with similar results. The graph represents the fold changes in mean ± SD of more than three independent experiments performed in triplicate. *P<0.05, **P<0.01 when compared with rotenone-or MPO-treated cells. B. Primary microglia were pretreated with or without 10 µM resveratrol for 1 h before exposure to 30 nM rotenone for 3 h, and then incubated with 5 µM DCF for 30 min at 37°C. DCF fluorescence was measured by FACS analyses. The graph represents the fold changes in MFI ± SD from four independent experiments. *P<0.05, **P<0.01 when compared with rotenone-treated cells. C. Primary microglia were pretreated with 10 µM resveratrol for 1 h, and then treated with 30 nM rotenone for 24 h. The intracellular levels of gp91 phox were determined by Western blot analyses. The bar graph represents quantitative analysis of protein band intensity from three independent experiments using ImageJ. **P<0.01, ***P<0.001 when compared with rotenone-treated cells.

To further address the effects of resveratrol on MPO-triggered inflammatory responses, we examined the expression levels of several inflammatory mediators in rat primary microglia. Reverse transcription-polymerase chain reaction (RT-PCR) analyses and quantitative real-time PCR analyses showed that pretreatment of microglia with resveratrol markedly suppressed the rotenone-induced expression of representative pro-inflammatory mediators, including tumor necrosis factor-alpha (TNF-α), cyclooxygenase-2 (COX-2), and inducible nitric oxide synthase (iNOS), in rat primary microglia (Fig. 5A, upper). Similar effects of resveratrol were observed in MPO-exposed primary microglia (Fig. 5A, lower). Next, we monitored the generation of intracellular ROS. The fluorescence intensity of the redox probe, CM-H_2_DCFDA (DCF), was considerably reduced in resveratrol-treated rat primary microglia compared to cells treated with rotenone alone (Fig. 5B), indicating that resveratrol attenuates the rotenone-induced increase in intracellular ROS. In addition, we examined the expression level of gp91phox, a subunit of NADPH oxidase that generates superoxide anion and is reported to be up-regulated in classically activated microglia under neurodegenerative conditions, including PD [36]. Western blot and FACS analyses showed that gp91 phox levels in primary microglia were increased by exposure to rotenone, an effect that was significantly reduced by resveratrol (Fig. 5C and data not shown). These results provide further support for the suppressive action of resveratrol on the production of inflammatory mediators in rotenone-exposed microglia. Taken together, these findings suggest that the down-regulation of MPO and absence of associated NO overproduction induced by resveratrol may contribute to attenuation of inflammatory responses in both rotenone- and MPO-exposed microglia.

### Resveratrol Relieves Impaired Responses to Rotenone in Primary Mixed Glial Cells from *Mpo^−/−^* Mice

**Figure 6:**
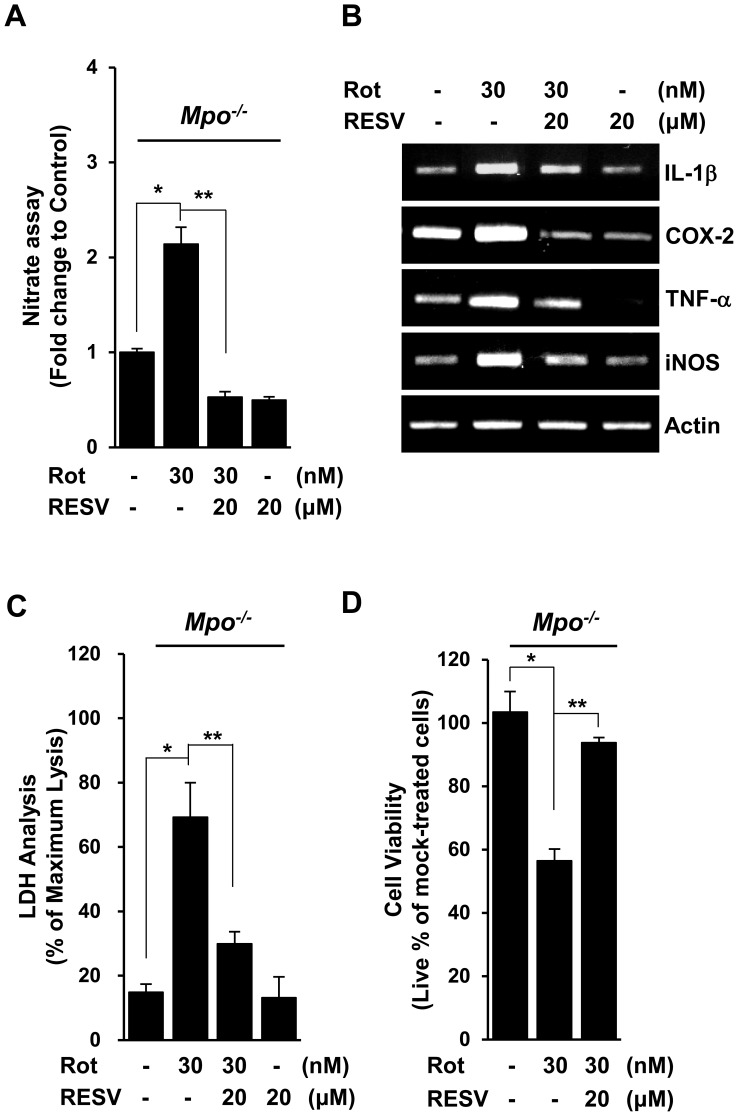
Resveratrol relieves the impaired inflammatory responses of MPO-deficient primary mixed glia to rotenone exposure. **A.** Primary mixed glial cells from *Mpo^−/−^* mice were mock-treated or treated with the indicated concentrations of resveratrol (RESV) for 1 h before exposure to 30 nM rotenone (Rot) for 24 h, and the supernatants were assayed for nitrate concentration. The results are the fold changes in mean ± SD of three experiments performed in triplicate.*P<0.05, **P<0.01 when compared with rotenone-treated cells. **B.** Primary mixed glial cells from *Mpo^−/−^* mice were mock-treated or treated with 20 µM resveratrol prior exposure to 30 nM rotenone. The levels of IL-1β, COX-2 and TNF-α mRNA in primary mixed glia from *Mpo^−/−^* mice were determined by RT-PCR-based analyses. The data are representative of more than three independent experiments. **C–D.** Primary mixed glial cells from *Mpo^−/−^* mice were incubated with the indicated concentrations of resveratrol and/or rotenone for 3 days. Cell viability was determined using the LDH assay (**C**) and the CCK-8 assay (**D**). Data were expressed as the percentage of cell death or cell viability to mock-treated cells from more than three independent experiments (**C** and **D**, respectively). *P<0.05, **P<0.01 when compared with rotenone-treated cells.

MPO-deficient mixed glial cells display impaired response to rotenone exposure, as evidenced by increased levels of inflammatory mediators and excessive cell death under rotenone-exposed conditions [11]. Accordingly, we determined whether resveratrol could relieve the impaired response of MPO-deficient mixed glial cells to rotenone exposure. Primary cultures of mixed glial cells from *Mpo^−/−^* mice were mock-treated or treated with rotenone in the presence or absence of the indicated concentrations of resveratrol for 1 day, and NO production was determined by measuring the amount of NO converted to nitrite in the media. Compared to MPO-deficient microglial cells treated with vehicle, NO release was increased in cells treated with rotenone; this increase was considerably diminished by treatment with resveratrol (Fig. 6A). In addition, increased basal NO level in MPO-deficient mixed glial cells was significantly reduced by resveratrol (data not shown). Furthermore, resveratrol significantly attenuated the rotenone-induced transcriptional up-regulation of several inflammatory mediators, including interleukin-1 beta (IL-1β), COX-2, TNF-α, and iNOS in MPO-deficient primary glial cells (Fig. 6B).

To extend the above results, we examined the effects of resveratrol on the viability of MPO-deficient mixed glia after exposure to rotenone. Primary cultures of mixed glial cells from *Mpo^−/−^* mice were incubated with rotenone in the presence or absence of resveratrol, after which the degree of cell death was determined by measuring lactate dehydrogenase (LDH) release into the media. As shown in Fig. 6C, the viability of mixed glia from MPO-deficient mice was reduced after exposure to rotenone, an effect that was significantly attenuated by treatment with resveratrol. Similar results were obtained by fluorescence microscopy using the CCK-8 assay (Fig. 6D). Taken together, these findings suggest that resveratrol alleviates the impaired response of MPO-deficient mixed glial cells to rotenone exposure through down-regulation of inflammatory mediators and abnormal increase in NO production, resulting in inhibition of excessive cell death.

### Rotenone-induced Neuronal Injury is Attenuated by Resveratrol in Neuron-microglia Co-cultures, but not in Neurons Cultured alone

**Figure 7:**
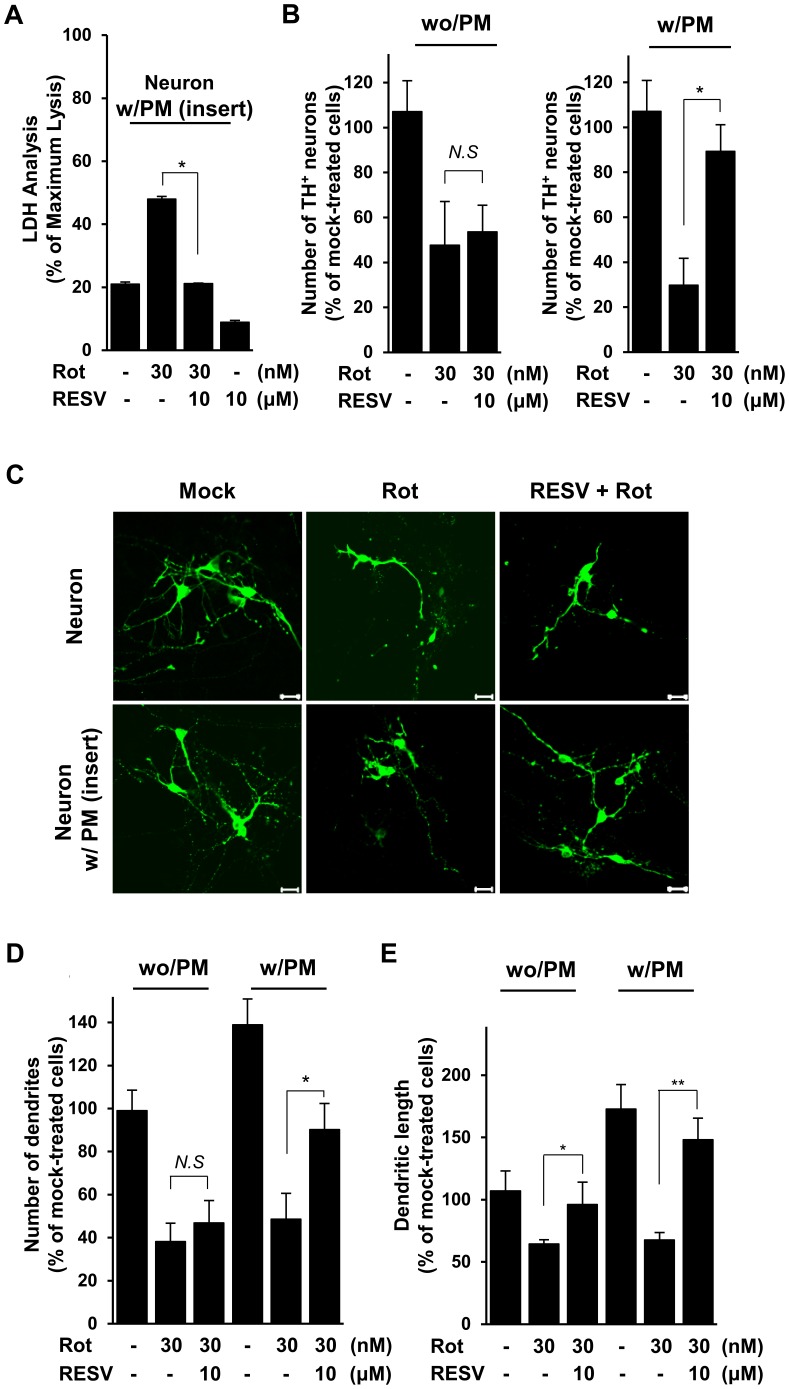
Rotenone-triggered neuronal injury was attenuated by resveratrol in neuron-microglia co-cultures. **A.** Rat primary mesencephalic neurons were incubated with or without rat primary microglia (PM) using transwell chambers, and the cells were treated with 10 µM resveratrol and/or 30 nM rotenone for 3 days. Cell viability was analyzed using the LDH assay. Data were expressed as the percentage of cell death relative to maximum LDH control. The results are the mean ± SD of three experiments performed in triplicate. ***P<0.001 when compared with rotenone-treated cell. **B.** TH-positive dopaminergic neurons were determined in rotenone- and/or resveratrol-treated rat primary mesencephalic cultures with or without primary microglia. The results for TH positive cells are expressed as a percentage of the mock-treated control cultures. *P = 0.002 when compared with rotenone-treated cell; *N.S.* no significant difference. **C-E.** Primary neuron-microglia co-cultures were treated with 30 nM rotenone and/or 10 µM resveratrol for 1 day. The morphological changes of dopaminergic neuronal cells were evaluated immunocytochemically using antibodies specific for TH (green) (**C**). Scale bar = 20 µm. The primary dendrite number (**D**) and maximal dendrite length (**E**) of TH-positive neurons were measured using TH immunocytochemistry and AxioVision software. The results expressed as a mean percent change ± SD of the mock-treated control values from three independent experiments. *P = 0.005, **P = 0.031, ***P = 0.002; *N.S.,* no significant difference.

Next, we addressed whether treatment of glial cells with resveratrol could confer protection against rotenone-induced neuronal injury. Accordingly, we measured the viability of rotenone-exposed neurons in the presence of microglia, with or without resveratrol. For this, rat primary mesencephalic neuron-enriched cultures were incubated with primary microglia using transwell chambers, and the cells were mock-treated or treated with rotenone in the presence or absence of resveratrol for 3 days. The extent of neuronal cell death was determined using LDH analyses and a CCK-8 kit (Fig. 7A and data not shown). Rotenone-induced neuronal cell death was considerably attenuated by treatment with resveratrol compared to that in neurons treated with rotenone alone (Fig. 7A), indicating that resveratrol alleviates rotenone-induced neuronal cell death in co-cultures with microglia.

Because PD is characterized by dopaminergic neuronal loss, we further examined whether resveratrol could influence dopaminergic neurons by immunostaining with an anti-tyrosine hydroxylase (TH) antibody. As shown in Fig. 7B, the number of TH-stained neurons was markedly decreased by exposure to rotenone in both the presence and absence of microglial cells. Notably, the rotenone-induced decrease in the number of TH-stained neurons was significantly attenuated by addition of resveratrol in co-cultures with primary microglia (Fig. 7B). However, we did not observe any significant effect of resveratrol on the number of TH-stained cells in neurons cultured alone. Furthermore, we found that treatment with resveratrol attenuated the rotenone-induced loss of dendrites and decrease in dendrite length in neuron-microglia co-cultures (Figs. 7C–E). These results suggest that resveratrol influences the glial response to rotenone via down-regulation of MPO and inflammatory mediators, and by extension, could alleviate neuronal injury in the rotenone-exposed brain.

### Similar Regulatory Effects of Resveratrol on MPO Levels is Observed in Microglia Treated with MPP^+^

**Figure 8:**
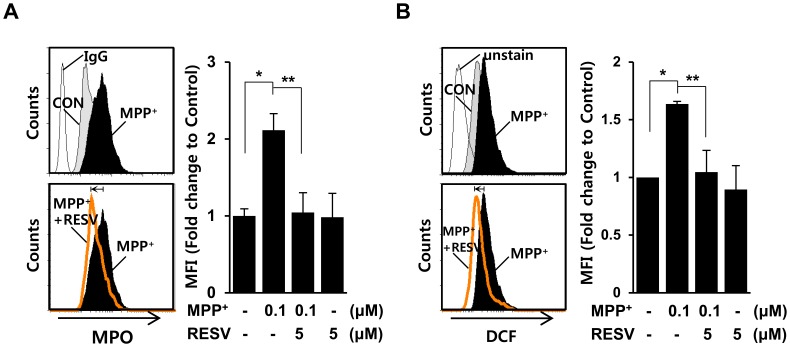
MPP^+^-induced MPO levels and ROS were reduced by resveratrol in microglia. **A.** BV2 microglia were treated with 0.1 µM 1-methyl-4-ph9enylpyridinium (MPP^+^) and/or 5 µM resveratrol (RESV) for 1 day, after which the intracellular levels of MPO were analyzed by FACS using an anti-MPO antibody. *P = 0.002, **P = 0.014 when compared with MPP^+^-treated cell. **B.** BV2 microglia were treated with 01 µM MPP^+^ and/or 5 µM resveratrol for 3 h, and then incubated with 5 µM DCF for 30 min at 37°C. DCF fluorescence was measured by FACS analyses. The data are representative of three independent experiments with similar results. The graph represents the fold changes in MFI (mean fluorescence intensity) ± SD from three independent experiments in triplicate. *P = 0.006, **P = 0.018 when compared with MPP^+^-treated cell.

To further delineate the effects of resveratrol on MPO levels in glial cells, we examined whether resveratrol could show regulatory effects on the MPO levels increased by other glial activators. We first searched for glial activators that could elevate MPO levels, and found that MPO levels were considerably increased by 1-methyl-4-phenylpyridinium (MPP^+^), a dopaminergic neurotoxin, which produces *in vivo* and *in vitro* cellular changes characteristic of PD. We then examined whether MPO levels could be changed by addition of MPP^+^ in microglia. BV2 cells were left untreated or pretreated with 5 µM resveratrol for 1 h, and mock-treated or treated with 0.1 µM MPP^+^. As shown in Fig. 8A, MPP^+^-stimulated increases of MPO levels were significantly attenuated by resveratrol. In addition, we observed that MPP^+^-induced production of ROS was also suppressed in the presence of resveratrol (Fig. 8B). These results further support the beneficial effects of resveratrol on MPO levels and neurodegenerative disease-associated inflammatory events in microglia.

## Discussion

Growing evidence indicates that environmental toxins such as pesticides could be important underlying risk factors for the development of numerous diseases. Rotenone, one of the most widely used herbicides, is a highly lipophilic compound that readily crosses the blood-brain barrier and accumulates throughout the brain [37]. Rotenone has specifically been suggested to provoke critical damage to mitochondrial function and oxidative system, ultimately leading to neurodegenerative diseases, including PD [38], [39]. Studies have shown that mice and rats given rotenone exhibit clinical and pathological features of PD, such as loss of TH-positive dopaminergic neurons and/or motor dysfunction [40]–[42]. In addition, epidemiological studies support the relationship between rotenone exposure and the development of PD in human populations [23]. However, the mechanisms underlying the harmful effects of rotenone in the brain are as yet poorly understood, and therapeutic approaches to PD remain an unmet need.

Recently, we found that a distinguishing feature of rotenone-exposed brain-resident immune cells is an increase in the activity and level of MPO that is undetectable under normal conditions [11]. Notably, our studies with *Mpo^−/−^* mice also revealed that MPO deficiency caused excessive generation of ROS in glia, suggesting that appropriate MPO activity-based glial responses could be necessary for efficient resolution of the rotenone-induced pathological state in the brain. These results have led us to investigate how regulation of MPO might be exploited to efficiently resolve rotenone-triggered pathological conditions in the brain. MPO has been shown to act as a metabolic “sink” for NO. Thus, we hypothesized that MPO-deficient glial cells could inappropriately regulate ROS and reactive nitrogen species, perhaps due to increase of NO bioavailability, thereby causing pathological outcomes under rotenone-exposed conditions. Therefore, we searched for therapeutic candidates that could reduce abnormal MPO activity without inducing aberrant NO generation, and examined their effects on activated glia and injured neuron under rotenone-exposed conditions. Based on the results of our previous experiments and a thorough literature search, we carefully selected the candidates that have been identified to reduce iNOS expression and NO release in glial cells, and then examined the effects of them on MPO activity and NO release in rotenone-treated glial cells. Interestingly, we found that resveratrol significantly reduced both MPO levels and NO production in rotenone-exposed microglia. In our *in vitro* experiment, we used 5–20 µM resveratrol, a concentration range previously shown to be effect *in vitro* [15], [43]. Interestingly, resveratrol appears to be able to concomitantly reduce both MPO activity and NO overproduction, thereby suppressing excessive NO release under MPO-deficient conditions. These studies thus demonstrate the novel finding that resveratrol has the ability to concurrently regulate MPO levels and NO production, affecting the activation state of glia under pathological conditions such as rotenone exposure.

Recent interesting reports have revealed that MPO not only has catalytic activity but also exhibits cytokine-like properties, directly activating and modulating inflammatory signaling cascades [44], [45]. To validate the effects of resveratrol on glia exposed to rotenone or MPO, we examined whether resveratrol could influence the cytokine-like properties of MPO in microglia. Resveratrol markedly suppressed phagocytic activity as well as expression of inflammation-associated genes in microglia exposed to either rotenone or MPO. Because MPO deficiency in glia potentiated, rather than reduced, the rotenone-stimulated generation of ROS and increased the levels of several inflammatory mediators compared with normal wild-type controls [11], we further examined the effects of resveratrol on primary mixed glia from *Mpo^−/−^* mice after rotenone exposure. Intriguingly, treatment of mixed glia from *Mpo^−/−^* mice with resveratrol significantly suppressed rotenone-induced expression of inflammation-associated genes as well as NO release. In addition, the viability of rotenone-exposed glia from *Mpo^−/−^* mice was considerably increased by addition of resveratrol. These observations suggest that resveratrol is able to efficiently modulate the levels of both MPO and NO, thereby influencing the activation state of glia.

PD is a neurodegenerative movement disorder that is characterized by dopaminergic neuronal loss in the substantia nigra [22]. Our previous study showed that rotenone-induced neuronal cell death in co-cultures with mixed glia from *Mpo^−/−^* mice was significantly increased compared to that seen in co-cultures with glia from wild-type mice, suggesting the importance of MPO activity in glia under rotenone-exposed conditions [11]. Moreover, we did not detect any meaningful increase in MPO levels or activity in rotenone-exposed neurons. Thus, we examined whether resveratrol influenced the properties of mesencephalic neurons in co-cultures with glia, and found that it significantly attenuated rotenone-induced neuronal cell death. Similar results were obtained by immunostaining with dopaminergic neuron-specific tyrosine hydroxylase (TH). In addition, confocal microscopy showed that TH-stained dopaminergic neurons in co-cultures with microglia treated with resveratrol developed dendrites that were longer and more elaborate than those in mock-treated cultures. Moreover, we found that MPP^+^ (1-methyl-4-phenylpyridinium), a neurotoxin known to cause PD in experimental animals and humans, increased MPO levels in microglia, which also considerably suppressed by resveratrol. In addition, animal studies have shown that orally administered resveratrol can cross the blood brain barrier and accumulate in the cerebral cortex [21], [46], [47]. These findings further support that resveratrol confers protective effects on rotenone-exposed neurons, and strengthen the possible impact of resveratrol for the PD filed.

It has been suggested that resveratrol shows inhibitory effects on LPS-triggered inflammatory events in microglia [16], [48]. In particular, Zhang *et al.* have recently reported that resveratrol protects DA neurons against LPS-induced neurotoxicity through inhibiting the activation of microglia [16]. We also observed the potent inhibitory effects of resveratrol on LPS-induced NO release in primary microglia. However, we did not observe not only a marked increase of MPO and but also regulatory effects of resveratrol on MPO levels in LPS-treated microglia and astrocytes (data not shown). Based on previous reports and our findings, resveratrol appears to have potent protective effects on activation of glia and neurotoxicity in multiple ways.

In recent years, considerable attention has focused on the importance of glial function in the context of a pathological CNS environment [34], [49]. Inappropriate glial activation contributes to pathological outcomes, such as neurodegenerative disease [30], [36], [50]. In this study, we provide the novel finding that concurrent down-regulation of MPO levels and NO release confer on glial ability to effectively and efficiently fight rotenone exposure, and thereby suppress neuronal damage. In addition, our data suggest that the natural compound resveratrol suppresses not only rotenone-induced increase of MPO but also aberrant NO production under MPO-deficient conditions in glia. Collectively, these findings further expand our current understanding of the characteristics of MPO and its regulatory mechanism under inflammatory conditions in the brain, and provide a new range of therapeutic applications for resveratrol as an efficient MPO modulator in diseases associated with abnormal MPO levels.

## Supporting Information

Figure S1 **Resveratrol regulates MPO levels in microglia and astrocytes. A.** Rat primary microglia (PM, up) and astrocytes (PA, lower) were pretreated with resveratrol (RESV), ethyl pyruvate (EtPy), or 15d-PGJ2 for 1 h, followed by incubation with 100 ng/ml MPO for 18 h. MPO levels were determined by Western blot analyses. **B.** Concentration-dependent effects of resveratrol were observed in MPO-treated primary microglia. **C.** Rat primary astrocytes were treated with 20 µM resveratrol and/or 100 ng/ml MPO at various time points. The cells were further incubated for 18 h and then MPO levels were determined by western blot. The bar graph represents quantitative analysis of protein band intensity from three independent experiments using ImageJ. *P<0.05, **P<0.01 when compared with MPO-treated cells; N.S., no significant difference.
(PDF)

Table S1 **Summary of statistical results presented in this study.**
(PDF)
