# Supplementary figures and images for: Resveratrol Confers Protection against Rotenone-Induced Neurotoxicity by Modulating Myeloperoxidase Levels in Glial Cells
Source: PLoS One. 2013 Apr 8;8(4):e60654. doi: 10.1371/journal.pone.0060654 (PMC3620483; doi:10.1371/journal.pone.0060654)

Supporting Information (Figure S1)

A

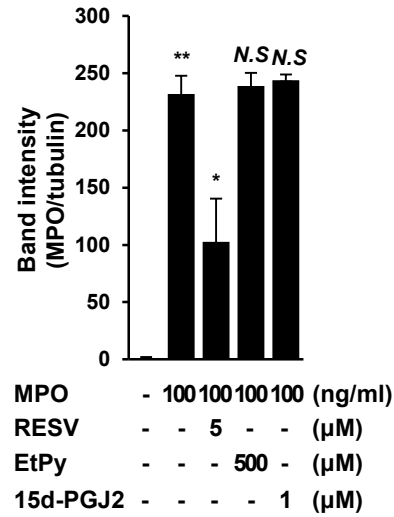

B

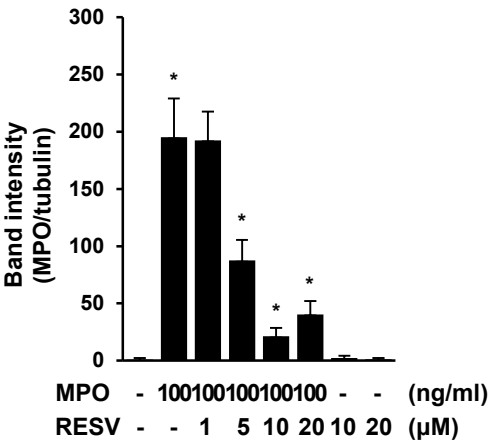

C

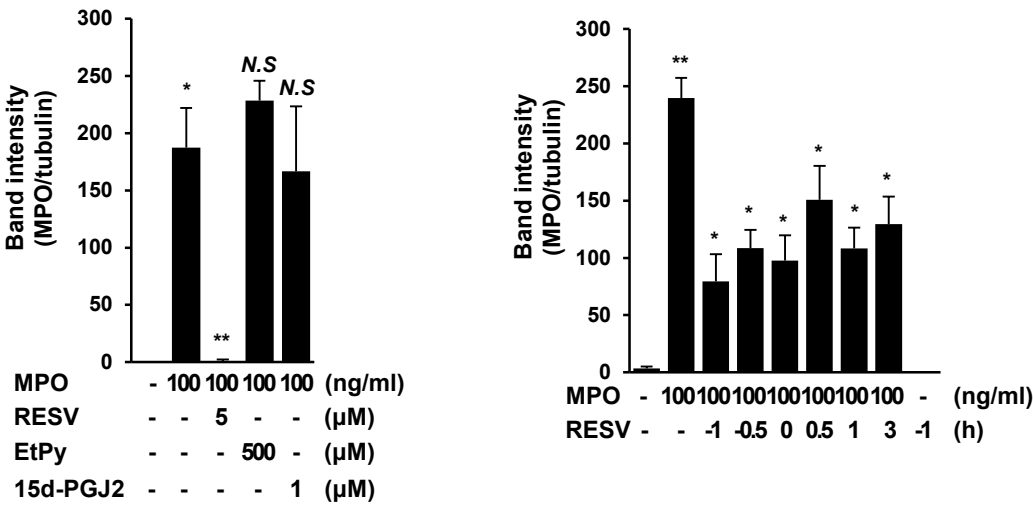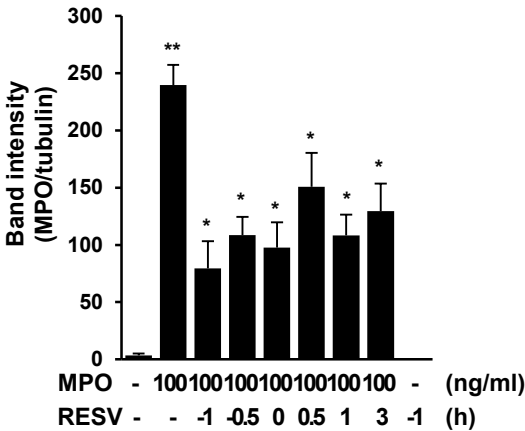

Supplement: Figure S1 — Resveratrol regulates MPO levels in microglia and astrocytes. A. Rat primary microglia (PM, up) and astrocytes (PA, lower) were pretreated with resveratrol (RESV), ethyl pyruvate (EtPy), or 15d-PGJ2 for 1 h, followed by incubation with 100 ng/ml MPO for 18 h. MPO levels were determined by Western blot analyses. B. Concentration-dependent effects of resveratrol were observed in MPO-treated primary microglia. C. Rat primary astrocytes were treated with 20 µM resveratrol and/or 100 ng/ml MPO at various time points. The cells were further incubated for 18 h and then MPO levels were determined by western blot. The bar graph represents quantitative analysis of protein band intensity from three independent experiments using ImageJ. *P<0.05, **P<0.01 when compared with MPO-treated cells; N.S., no significant difference. (PDF) [file pone.0060654.s001.pdf]
