# Supplementary material for: Resveratrol Confers Protection against Rotenone-Induced Neurotoxicity by Modulating Myeloperoxidase Levels in Glial Cells
Source: PLoS One. 2013 Apr 8;8(4):e60654. doi: 10.1371/journal.pone.0060654 (PMC3620483; doi:10.1371/journal.pone.0060654)
Supplement: Table S1 — Summary of statistical results presented in this study. (PDF) [file pone.0060654.s002.pdf]

Supporting Information (Table S1)

Figures		Comparing Groups	df	F value	P value	Symbol
Figure 1	A	Control vs. Rotenone	1	47.755	0.003	**
		Rotenone vs. Rotenone + Resveratrol	1	20.965	0.01	*
	B	Control vs. Rotenone	1	10.446	0.032	*
		Rotenone vs. Rotenone + Resveratrol	1	23.135	0.009	**
	C	Control vs. Rotenone	1	11.138	0.01	*
		Rotenone vs. Rotenone + Resveratrol 5 μM	1	32.999	0.001	**
		Rotenone vs. Rotenone + Resveratrol 10 μM	1	146.596	0.001	**
Figure 2	A	Control vs. Myeloperoxidase	1	6.056	0.027	*
		Myeloperoxidase vs. Myeloperoxidase + Resveratrol	1	4.9	0.049	*
	C	Control vs. Myeloperoxidase	1	75.811	< 0.001	***
		Myeloperoxidase vs. Myeloperoxidase + Resveratrol 5 μM	1	5.745	0.038	*
		Myeloperoxidase vs. Myeloperoxidase + Resveratro 10 μM	1	10.353	0.009	**
		Myeloperoxidase vs. Myeloperoxidase + Resveratro 20 μM	1	55.129	< 0.001	***
	D (*left*)	Control vs. Myeloperoxidase	1	20.285	0.002	**
		Myeloperoxidase vs. Myeloperoxidase + Resveratrol 5 μM	1	6.243	0.041	*
		Myeloperoxidase vs. Myeloperoxidase + Resveratro 10 μM	1	16.988	0.002	**
		Myeloperoxidase vs. Myeloperoxidase + Resveratro 20 μM	1	13.038	0.007	**
	D (*right*)	Myeloperoxidase vs. Myeloperoxidase + ABAH	1	15.848	0.002	**
		Myeloperoxidase vs. Myeloperoxidase + SHA	1	15.74	0.002	**
Figure 4	A (*a*)	Control vs. Rotenone	1	29.135	0.006	**
		Rotenone vs. Rotenone + Resveratrol	1	19.58	0.012	*
	A (*b*)	Control vs. Myeloperoxidase	1	57.076	0.002	**
		Myeloperoxidase vs. Myeloperoxidase + Resveratrol	1	83.751	< 0.001	***
	B	Control vs. Rotenone	1	75.763	< 0.001	***
		Rotenone vs. Rotenone + Resveratrol	1	83.751	< 0.001	***
	C	Control vs. Myeloperoxidase	1	10.648	0.01	*
		Myeloperoxidase vs. Myeloperoxidase + Resveratrol	1	13.395	0.007	**
Figure 5	A (*upper left*)	Control vs. Rotenone	1	80.454	0.001	**
		Rotenone vs. Rotenone + Resveratrol	1	48.623	0.002	**
	A (*upper right*)	Control vs. Rotenone	1	6.004	0.044	*
		Rotenone vs. Rotenone + Resveratrol	1	12.977	0.011	*
	A (*lower left*)	Control vs. Myeloperoxidase	1	9.09	0.017	*
		Myeloperoxidase vs. Myeloperoxidase + Resveratrol	1	9.369	0.014	*
	A (*lower right*)	Control vs. Myeloperoxidase	1	29.57	0.006	**
		Myeloperoxidase vs. Myeloperoxidase + Resveratrol	1	12.252	0.025	*
	B	Control vs. Rotenone	1	6.814	0.035	*
		Rotenone vs. Rotenone + Resveratrol	1	31.457	0.002	**
	C	Control vs. Rotenone	1	26.592	< 0.001	***
		Rotenone vs. Rotenone + Resveratrol	1	17.479	0.006	**
Figure 6	A	Control vs. Rotenone	1	9.356	0.022	*
		Rotenone vs. Rotenone + Resveratrol	1	19.272	0.0019	**
		Rotenone vs. Resveratrol	1	24.272	0.002	**
	C	Control vs. Rotenone	1	10.607	0.014	*
		Rotenone vs. Rotenone + Resveratrol	1	13.084	0.011	*
	D	Control vs. Rotenone	1	29.425	0.002	**
		Rotenone vs. Rotenone + Resveratrol	1	7.576	0.033	*
Figure 7	A	Rotenone vs. Rotenone + Resveratrol	1	100.157	< 0.001	***
	B (*left*)	Rotenone vs. Rotenone + Resveratrol	1	0.042	0.846	N.S.
	B (*right*)	Rotenone vs. Rotenone + Resveratrol	1	26.181	0.002	**
	D	Rotenone vs. Rotenone + Resveratrol wo/PM	1	3.136	0.115	N.S.
		Rotenone vs. Rotenone + Resveratrol w/PM	1	33.053	0.005	**
	E	Rotenone vs. Rotenone + Resveratrol wo/PM	1	8.807	0.031	*
		Rotenone vs. Rotenone + Resveratrol w/PM	1	57.317	0.002	**
Figure 8	A	Control vs. MPP^+	1	97.974	0.002	**
		MPP^+ vs. MPP^+ + Resveratrol	1	13.886	0.014	*
	B	Control vs. MPP^+	1	28.43	0.006	**
		MPP^+ vs. MPP^+ + Resveratrol	1	14.958	0.018	*

A One-Way Analysis of Variance (ANOVA) was used to compare means of two groups. P value in figures are marked with an asterisk. * *P <0.05,* ** *P <0.01,* *** *P <0.001, N.S.*, no significant difference.
